# The epithelial-mesenchymal transition and the cytoskeleton in bioengineered systems

**DOI:** 10.1186/s12964-021-00713-2

**Published:** 2021-03-10

**Authors:** Susan E. Leggett, Alex M. Hruska, Ming Guo, Ian Y. Wong

**Affiliations:** 1 Department of Chemical and Biological Engineering, Princeton University, William St, Princeton, NJ 08544 USA; 2 School of Engineering, Center for Biomedical Engineering, and Joint Program in Cancer Biology, Brown University, 184 Hope St Box D, Providence, RI 02912 USA; 3 Department of Mechanical Engineering, MIT, 77 Massachusetts Ave, Cambridge, MA 02139 USA

**Keywords:** Actin, Vimentin, Cytoskeleton, Collective migration, Extracellular matrix

## Abstract

The epithelial-mesenchymal transition (EMT) is intrinsically linked to alterations of the intracellular cytoskeleton and the extracellular matrix. After EMT, cells acquire an elongated morphology with front/back polarity, which can be attributed to actin-driven protrusion formation as well as the gain of vimentin expression. Consequently, cells can deform and remodel the surrounding matrix in order to facilitate local invasion. In this review, we highlight recent bioengineering approaches to elucidate EMT and functional changes in the cytoskeleton. First, we review transitions between multicellular clusters and dispersed individuals on planar surfaces, which often exhibit coordinated behaviors driven by leader cells and EMT. Second, we consider the functional role of vimentin, which can be probed at subcellular length scales and within confined spaces. Third, we discuss the role of topographical patterning and EMT via a contact guidance like mechanism. Finally, we address how multicellular clusters disorganize and disseminate in 3D matrix. These new technologies enable controlled physical microenvironments and higher-resolution spatiotemporal measurements of EMT at the single cell level. In closing, we consider future directions for the field and outstanding questions regarding EMT and the cytoskeleton for human cancer progression.

**Video Abstract**

**Supplementary Information:**

The online version contains supplementary material available at 10.1186/s12964-021-00713-2.

## Background

The epithelial-to-mesenchymal transition (EMT) occurs when tightly connected epithelial cells acquire a migratory mesenchymal phenotype during embryonic development, wound healing, and disease [1, 2]. Historically, EMT has been associated with a profound reorganization of the cytoskeleton in order to weaken cell–cell attachment and strengthen cell–matrix adhesions [3], and was first observed by Elizabeth Hay in response to instructive cues from the extracellular matrix [4]. Indeed, tumor invasion and drug resistance are associated with dysregulated extracellular matrix, with aberrant matrix deposition and remodeling resulting in enhanced stiffness [5]. Cytoskeletal elements are well established as EMT biomarkers, particularly intermediate filaments such as keratin (in epithelial cells) and vimentin (in mesenchymal cells) [6]. Nevertheless, the functional importance of EMT and associated cytoskeletal changes remains poorly understood, particularly for cancer progression in humans [7]. Recent bioengineering innovations have enabled biomimetic assays and higher-resolution measurement tools to elucidate EMT at the single cell level over space and time.

Classical cell migration assays are based on experimental conditions that can artificially bias towards epithelial or mesenchymal states [8]. For example, “wound-healing” assays occur on rigid tissue culture plastic, based on the collective migration of tightly-connected epithelial monolayer sheets [9]. Alternatively, Transwell (Boyden) assays are based on a plastic microporous membrane, which cells must traverse as individual cells, impeding collective migration [10]. Tri-dimensional (3D) culture conditions, based on embedding cells within a compliant biomaterial, represent a promising alternative for investigating invasion and EMT [11, 12]. In particular, mammary epithelial cells cultured in reconstituted basement membrane (i.e. Matrigel) recapitulate differentiated tissue-like architectures with lumens and cell–cell junctions, which progressively disorganize and disseminate in response to aberrant microenvironmental cues [13]. More recently, engineered biomaterials [14] and microfabricated devices [15] have provided increased control over material stiffness, degradability, and architecture. These controlled microenvironmental conditions may provide new insights into how cell–matrix interactions mediate collective and individual migration phenotypes.

Phenotypic heterogeneity and plasticity are defining features of cancer cells, and remain challenging to investigate using bulk analyses at endpoints [16]. EMT is believed to occur rarely in a small subpopulation of cells, which may be overlooked without comprehensive single cell measurements. Live cell imaging with high spatial and temporal resolution may enable new insights into molecular and cellular-scale dynamics during invasion and EMT [17]. For instance, cytoskeletal protrusions are particularly important for directed migration, and cells can appreciably deform their surrounding ECM [18]. In turn, cells may undergo substantial deformations to traverse ECM, which may be facilitated by a more compliant cytoskeleton. It has been further hypothesized that cancer cells are significantly softer than their nontransformed counterparts, particularly in the context of stem-like states that express vimentin [19]. Overall, there is increased interest in subcellular resolution of cell–matrix adhesions [20], as well as collective behaviors mediated by cell–cell junctions [21].

Here, we review recent developments in EMT and the cytoskeleton in cancer (particularly intermediate filaments) enabled by biomimetic materials and higher resolution measurement technologies. We focus on publications within the last 5 years, and emphasize potential links between the mechanobiology of the intracellular cytoskeleton and the extracellular matrix. For more comprehensive treatments of EMT in cancer, we refer readers to other recent reviews on this topic [1–3, 7, 22]. We first provide a brief primer into the biochemistry and mechanics of the cytoskeleton as well as operational definitions for EMT. Next, we consider EMT-like behaviors during collective and individual migration on planar substrates. We further consider the dynamics of vimentin and EMT in 2D monolayer and 3D matrix culture. We then address the effect of submicron topographies (“2.5D”) and compliant 3D matrix on migration and EMT. Finally, we provide our perspective on EMT and cancer metastasis, as well as future directions for the field.

## Functional definitions for the epithelial-mesenchymal transition

**Fig. 1:**
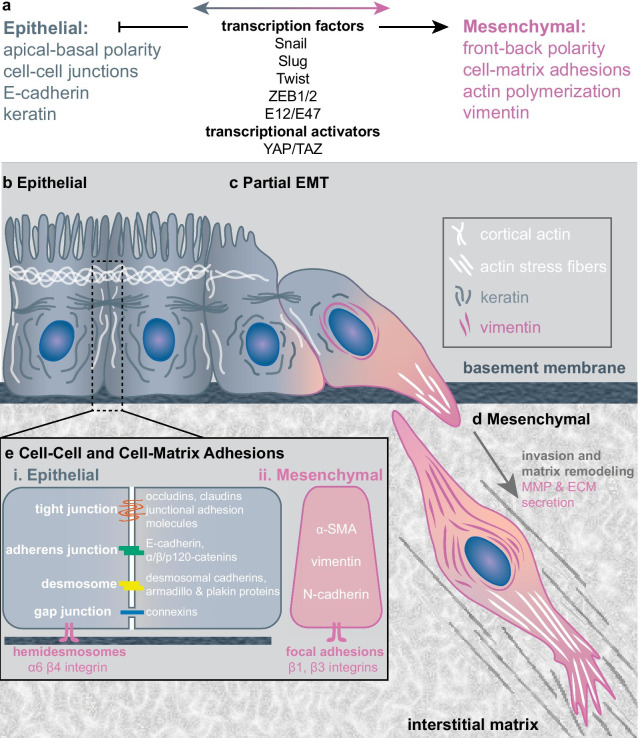
Schematic of EMT progression. **a** Common features of epithelial and mesenchymal phenotypes, as well as transcription factors that drive EMT. Artistic representation of cell morphology and cytoskeletal organization for **b** epithelial, **c** “partial” EMT, and **d** mesenchymal states. **e** Detailed depiction of cell–cell and cell–matrix adhesions

Classically, EMT has been understood as a multifaceted program of phenotypic changes that cause an epithelial cell to acquire mesenchymal characteristics, including altered polarity and cytoskeletal organization [22]. EMT programs can be activated by inflammatory stimuli including growth factors (e.g. TGF-*β*, HGF, EGF, WNT), hypoxia, and extracellular matrix components (e.g. collagen I), which act through developmental transcription factors (e.g. SNAIL, SLUG, TWIST, ZEB1/2, and E2A proteins, E12/E47) to repress E-cadherin expression and induce mesenchymal gene expression (Fig. 1a) [1]. Small, noncoding single-stranded RNAs (microRNAs or miRNAs) work in concert with transcription factors to regulate the promotion or repression of EMT signaling networks in a context-dependent manner [1]. For instance, the well-studied miR-34 and miR-200 miRNA families serve dual roles as tumor and EMT suppressors though the formation of double-negative feedback loops with SNAIL and ZEB1/2, respectively [23]. EMT may be activated to varying extents, yielding a diverse spectrum of intermediate states (“partial EMT”) and may be reversible through mesenchymal-to-epithelial transitions (MET). Altogether, these dynamic processes have been more broadly defined as “epithelial-mesenchymal plasticity” [2].

Epithelial tissues consist of sheet-like architectures that cover body surfaces and hollow organs (e.g. skin, breast, lung, colon, prostate, etc.) [24]. Cancers associated with epithelial tissues (i.e. carcinomas) represent the most common type of cancer in humans [25]. Epithelial cells typically organize into tightly-connected multicellular layers with strong cell–cell junctions on both lateral sides (e.g. tight junctions, adherens junctions, and desmosomes), which maintain apicobasal polarity and limit motility (Fig. 1b, e). In turn, these junctions are linked to the cytoskeleton, including a circumferential F-actin belt as well as keratins [26, 27]. Cortical F-actin is also distributed at the periphery of epithelial cells, while keratins form an extended network from the nuclear envelope out to the cell membrane. It should be noted that this cytoskeletal organization is maintained by cell–matrix adhesions anchored to the basement membrane.

YAP/TAZ transcriptional activators have also emerged as sensors of these cell–cell and cell–matrix interactions, which can trigger EMT [28] as well as stem-like phenotypes [29]. YAP/TAZ are part of the Hippo pathway, which is a key regulator of tissue homeostasis via growth control [30]. For instance, high cell densities or soft matrix are associated with inactive YAP/TAZ via localization in the cytoplasm or controlled degradation, which prevents proliferation in normal cells [29]. However, lower cell densities or stiffer matrix permits increased actin contractility, resulting in active YAP/TAZ via translocation to the nucleus to promote proliferation. Dysregulation of YAP/TAZ signaling is associated with loss of sensitivity to mechanical cues and uncontrolled proliferation in tumors. Moreover, YAP/TAZ activation has been associated with enhanced migration, drug resistance, anoikis-resistance in circulation, metabolic adaptation, and metastatic colonization (reviewed in [31]). YAP/TAZ further exhibit crosstalk with growth factor signaling (e.g. WNT, TGF-*β*, Hedgehog, EGF, Notch), as well as with various ligands associated with G-protein coupled receptors (GPCR) [30]. YAP/TAZ activation occurs dynamically in response to external mechanical and chemical cues, and an improved understanding of this signaling pathway requires the capability to perturb cells with a time-varying stimulus while reading out the phenotypic response over time.

Collective cell migration is increasingly recognized as a crucial mode of cancer invasion, and is associated with partial connectivity between migratory cells [8]. So-called “leader cells” retain some cell–cell junctions at their trailing edge, which allows them to mechanically coordinate the migration of “follower” cells (Fig. 1c) [32]. This phenomenon has been associated with a “partial” EMT, since cells may co-express epithelial and mesenchymal biomarkers (e.g. E-cadherin, keratins, vimentin), although operational definitions remain unresolved [7]. It should be noted that analogous processes occur during collective migration and EMT in embryonic development, which may provide useful biological insights [33].

Finally, a complete EMT occurs when initially epithelial cells undergo cytoskeletal organization with a loss of apicobasal polarity and gain of front-back polarity (Fig. 1d) [22]. In particular, cells weaken their cell–cell junctions and redistribute *β*-catenin, while forming actin-rich protrusions at the leading edge [3]. Moreover, cells gain expression of the intermediate filament vimentin, which is also distributed throughout the cell interior and protects the nucleus [34]. Classically, EMT is associated with total detachment of individual mesenchymal cells that exhibit elongated morphology and expression of vimentin, N-cadherin, and *α*-smooth muscle actin (Fig. 1e). Although a switch-like transition between tightly connected epithelial tissues and dispersed individual mesenchymal cells occurs in embryonic development and wound healing, the presence of these EMT transcription factors and biomarkers does not appear to be required for cancer metastasis in humans [7].

## Structure and function of the cytoskeleton

The cytoskeleton allows mammalian cells to resist deformation and coordinates dynamic force-generation for morphological changes and migration [18]. Recent measurements have yielded new insights into the functional role of various cytoskeletal proteins under extreme mechanical stresses. In general, polymerized cytoskeletal filaments tend to be relatively straight (i.e. rigid relative to thermal fluctuations) and are comparable in size to mammalian cells [35]. As a consequence, these semiflexible polymers can become physically entangled as a gel at relatively low concentrations (compared to more flexible synthetic organic polymers) [36]. Reconstituted networks of cytoskeletal filaments also exhibit counterintuitive strain stiffening, meaning that they strengthen under large deformations [37]. Such strain stiffening has been theoretically explained as an entropic mechanism, whereby straightening out a polymer suppresses thermal fluctuations and effectively increases mechanical resistance to deformation [38]. Alternatively, an enthalpic stretching model is based on preferential alignment of filaments in the direction of deformation, so that mechanical resistance is dominated by filament stretching rather than bending [39].

**Fig. 2:**
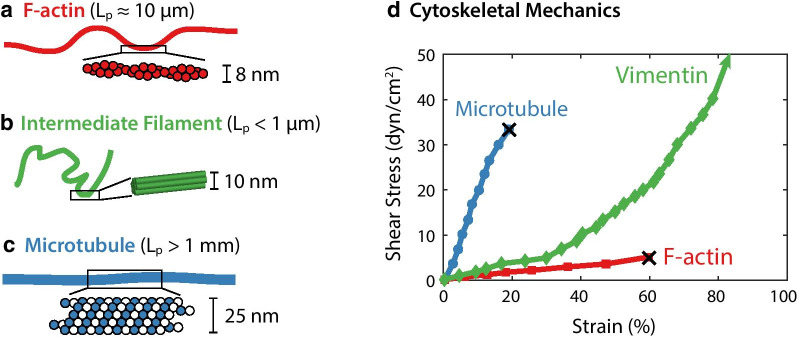
Cytoskeletal proteins and mechanics. Representative dimensions and structures of F-actin (**a**), intermediate filaments (**b**), and microtubules (**c**), inspired by Pegoraro et al.  [35]; “$L_p$” denotes persistence length. **d** Mechanical properties of reconstituted microtubule, vimentin intermediate filament, and F-actin networks, based on strain response under applied shear stress. “X” denotes mechanical rupture of network. Replotted from Janmey et al. [40]

Three types of cytoskeletal polymers are generally found in mammalian cells: filamentous actin (F-actin), microtubules, as well as intermediate filaments (Fig. 2) [18]. F-actin and microtubules are highly conserved throughout cell types and across animal species; they have also been widely studied in a variety of in vitro and in vivo systems. Both F-actin and microtubules exhibit a structural polarity that results in preferential polymerization at one end, which can generate protrusive forces that drive changes in cell shape. F-actin and microtubule networks are tightly regulated by a host of binding proteins that initiate or terminate monomer addition, disassemble or sever filaments, as well as organize filaments into higher-order crosslinked and oriented architectures [26].

### F-actin in cell polarity and directed migration

F-actin is somewhat flexible as an individual polymer, with a characteristic persistence length $L_p \approx$ 10 $\upmu$m (Fig. 2a, d) [41]. Nevertheless, F-actin can be further organized into mechanically reinforced architectures such as cylindrical bundles or space-filling networks [26]. F-actin polymerization can be spatially coordinated along the cellular periphery; F-actin polymerization in a bundled state drives localized cellular protrusions (i.e. filopodia), while branched network assembly over a broader leading edge advances lamellipodia [3]. F-actin can also be assembled with myosin motor proteins in an anti-parallel configuration to form stress fibers, which enable cells to apply contractile tractions to the extracellular matrix [42].

During directed cell migration, these processes act in sequence to translocate the cell body across a 2D surface or 3D matrix [43], regulated by RHO GTPases [44]. In particular, RHOA activation stimulates focal adhesion formation and actomyosin contractility, which can occur in response to TGF-*β* stimulation [45]. Subsequently, Rho-associated kinase (ROCK) signaling can promote actin polymerization via the formin diaphanous 1 (DIA1), along with inhibition of the actin stabilizing factor cofilin via LIM kinase (LIMK) [46], which have been recently shown to be crucial for strong protrusions in 3D matrix [47].

The transition from apicobasal polarity to front-rear polarity further occurs via crosstalk between RHO GTPases and polarity proteins (e.g. Crumbs, PAR, and Scribble) [48]. Breakdown of adherens junctions can translocate E-cadherin and beta-catenin from the cell surface to the cytoplasm [3], activating p120 catenin to locally repress RHOA activity [49]. Moreover, PAR and Scribble complexes will relocate to the leading edge of the cell, activating RAC1 and CDC42 for actin polymerization and membrane protrusion formation [50]. Local RAC1 activation can stimulate PI3K, which promotes microtubule polymerization that drives positive feedback to further stabilize RAC1 [51]. At the cell surface, EMT downregulates integrin $\alpha _6\beta _4$ that mediates adhesion to laminins in the basement membrane [52], and upregulates integrin $\alpha _5\beta _1$ to adhere to fibronectin [53], as well as integrin $\alpha _1\beta _1$ which binds to collagen I (Fig. 1e) [54, 55]. This process can also include an intermediate step of matrix remodeling via localized proteolysis (e.g. matrix metalloproteinases MMP2, MMP9, [56]) after integrin binding [57], although cells are also capable of “squeezing” forward using a propulsive amoeboid phenotype (which may not require matrix remodeling).

### Intermediate filaments and EMT

Intermediate filaments relevant to EMT include vimentin (a biomarker for mesenchymal phenotype) [34], as well as keratin (a biomarker for epithelial phenotype, also known as cytokeratin) [58]. Intermediate filaments tend to be relatively flexible ($L_p<$ 1 $\upmu$m) [59] and resist tensile forces more effectively than compressive forces (Fig. 2b) [40]. Intermediate filaments assemble laterally, whereby two monomers associate via their central domains to form parallel helical coils around each other [60]. Two dimers associate with each other in an antiparallel arrangement to form a staggered tetramer. The lateral association of multiple tetramers results in the formation of a unit-length filament (ULF). Subsequent longitudinal annealing of ULFs results in filament elongation, which is followed by radial compaction to achieve the final intermediate filament diameter. In contrast to F-actin organization, this assembly mechanism results in a polymer that lacks directional polarity and does not interact specifically with molecular motors. Nevertheless, as a consequence of this winding, rope-like organization, intermediate filaments can withstand large deformations without breaking (Fig. 2d). Indeed, in vitro experiments found that reconstituted networks of both vimentin and keratin networks are predominantly elastic, with a strong nonlinear stiffening behavior [61]. Similar to vimentin networks, keratin networks (e.g. K8/K18, K5/K14) show an elastic behavior under bulk rheology, with a weak frequency dependence [62, 63]. Interestingly, keratin networks have comparable mechanical properties as vimentin networks, with a shear storage modulus $G' \sim$ 1 Pa at a concentration of $\approx 1$ mg/mL. The addition of divalent cations such as Ca$^{2+}$ and Mg$^{2+}$ can act as crosslinkers that stiffen vimentin or keratin networks [64], and can also facilitate bundle formation [65]. This is highly relevant to the regulation of cell mechanics since the concentrations of divalent cations in mammalian cells often change drastically in space and time [66]. One caveat of these reconstituted intermediate filament networks is that they are not phosphorylated as they would be in the mammalian cytoskeleton, since they are often prepared by recombinant expression in bacteria (which lacks homologs of these intermediate filament proteins). This lack of phosphorylation is likely to affect intermediate filament assembly, network architecture, and mechanics. Thus, recent efforts have focused more directly on probing the mechanics of IF networks in situ in mammalian cells.

Intermediate filaments are thought to protect cells against extreme deformation, and genetic manipulation of vimentin or keratin is often deleterious [34]. It should be noted that keratin is more complex in its biological regulation compared to vimentin. Mutations that impair keratin intermediate filament assembly (e.g. K5, K14) result in mechanically fragile skin that blisters easily, known as epidermolysis bullosa simplex (EBS) [67]. In single keratinocytes, keratin depletion also results in greater deformability and invasion [68, 69], but is not sufficient for EMT in cell lines or mouse models [70, 71]. In comparison, genetic knockout of vimentin in mouse models resulted in impaired cell migration and wound healing [72, 73]. Overall, the functionality of intermediate filaments remains poorly understood, particularly since its dynamics are considerably slower than other cytoskeletal proteins (polymerizing in minutes with network remodeling on the order of hours).

### Microtubules and cytoskeletal crosstalk

Microtubules are the most rigid of cytoskeletal filaments, with a characteristic straightness (i.e. persistence length $L_p$) that exceeds 1 mm (Fig. 2c) [41]. Thus, microtubules exhibit nearly straight conformations within a cell and emanate radially outwards from the cell nucleus, analogous to a hub and spoke geometry. Although microtubules are relatively stiff, they can buckle under relatively small strains, which triggers rapid disassembly (Fig. 2d) [74]. This so-called dynamic instability has been proposed as a mechanism to efficiently search intracellular space for chromosomes, facilitating mitotic spindle formation during cell division.

Coordinated activities between cytoskeletal proteins are likely to enhance cell migration. Previous work has focused on individual cells (e.g. fibroblasts) in 2D culture. For instance, nascent focal adhesions driven by actin polymerization may be reinforced by vimentin through the cytoskeletal linker plectin [75]. Similarly, vimentin has been shown to directly interact with the actin-binding protein filamin A, which mediates integrin trafficking and activation [76]. Alternatively, vimentin and microtubules assemble cooperatively via APC, which acts to sustain cell polarity [77]. Furthermore, vimentin disassembly modulates actin-based lamellipodia, which also maintains an asymmetric morphology [78]. Moreover, the focal adhesion scaffold protein Hic-5 has been implicated in the regulation of vimentin networks in fibroblasts [79], as well as cell shape and invasion of breast cancer cells [80].

More recent work with cell monolayers in 2D culture showed that intermediate filaments can also sustain cell–cell contacts and bias cell–matrix adhesions [81]. The crosstalk between cytoskeletal components for 3D migration is less well understood, although all three cytoskeletal proteins have been shown to play a role in invadopodia-based protrusions through the basement membrane [82].

## Scattering and leading: new insights from old assays

**Fig. 3:**
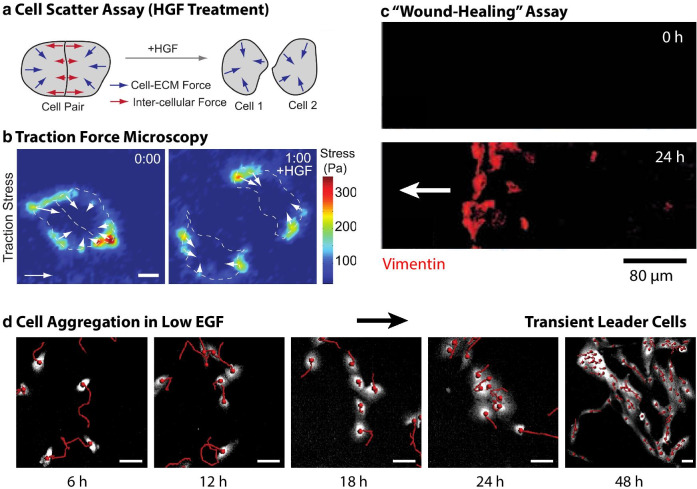
Scattering, wound-healing, and aggregation assays. **a** Scattering assays visualize how multicellular epithelial (MDCK) clusters disperse into migratory individuals in response to exogeneous biochemical stimulation (e.g. HGF). **b** Traction force microscopy reveals cell-generated forces on deformable substrates, which are localized at the cluster and cell periphery. Reproduced from [86] with permission. **c** Wound healing assays visualize the collective migration of sheet-like monolayers into unoccupied regions. Mammary epithelial (MCF-10A) cells at the migration front (wound edge) gain vimentin expression (vimentin immunolabeling, red), analogous to an EMT. Reproduced from Gilles et al. [88] with permission. **d** Mammary epithelial cells cultured with reduced levels of epidermal growth factor initially aggregate into multicellular clusters, analogous to a reverse mesenchymal-to-epithelial transition. GFP cytoplasm (white) with individual cell tracks (5h, red). Subsequently, leader cells transiently guide migration to link clusters together into spanning networks. Reproduced from [89] with permission

Cell scattering assays reveal how adherent epithelial clusters disperse into migratory individuals after exogenous treatment with soluble factors on planar 2D substrates, analogous to an EMT (Fig. 3a). For example, Madin-Darby canine kidney (MDCK) epithelial cells typically form isolated multicellular clusters with strong E-cadherin junctions, which disassemble after treatment with hepatocyte growth factor (HGF, “scatter factor”) [83, 84]. de Rooij et al. [85] subsequently showed that HGF treatment increases actomyosin contractility via integrin-mediated substrate adhesion, independent of E-cadherin expression. In order to directly visualize cell-generated forces, Maruthamuthu et al. plated MDCK cells on compliant polyacrylamide hydrogel substrates labeled with fluorescent tracer particles [86]. These MDCK clusters initially exhibited strong tractions around the periphery, as well as strong intercellular adhesions through E-cadherin (Fig. 3b). Upon HGF stimulation, some MDCK cells extended protrusions in the direction perpendicular to the cell–cell interface, resulting in a sudden rupture. In comparison, other MDCK cells extended protrusions in a direction parallel to the cell–cell interface, resulting in a gradual decrease in cell–cell adhesion before detachment. These results confirm that HGF does not directly weaken cell–cell junctions (e.g. by downregulation of E-cadherin), but rather mediates the mechanical crosstalk with the actin cytoskeleton and focal adhesions. Loerke et al. subsequently implemented image-based phenotypic screening of scattering MDCK cells, which identified a number of targeted inhibitors that acted on cell–cell adhesion, migration, or both [87].

So-called “wound healing” assays characterize how sheet-like monolayers migrate collectively to occupy empty regions of planar 2D substrates [9]. Gilles et al. [88] investigated vimentin expression in confluent mammary epithelial cells (MCF-10A) after applying controlled damage in a circular region using a drop of sodium hydroxide. Initially, vimentin expression was minimal in the confluent epithelial monolayer (Fig. 3c). After 24 h, vimentin expression occurred in a localized region near the migration front, associated with “leader cells” undergoing a (partial) EMT (Fig. 3c). By 8 days, vimentin expression was again minimal everywhere once cells had reestablished a confluent monolayer. Moreover, knockdown of vimentin expression impeded the migration of these leader cells and front closure. Similarly, vimentin expression and migration were attenuated for wounded cultures deprived of epidermal growth factor (EGF). Subsequently, Riahi et al. spatially profiled gene expression of leader cells in a different mammary epithelial cell line (MCF-7), revealing EMT occurred through Notch1-Dll4 signaling [90]. Subsequent research by Reffay et al. [91] demonstrated that these leader cells mechanically coordinate followers through actomyosin contractility acting through cell–cell junctions.

Leggett et al. [89] showed that mammary epithelial cells in reduced EGF exhibit multicellular aggregation into clusters (analogous to a reverse MET), followed by leader cell formation. Since nontransformed mammary epithelial cells (MCF-10A) are growth factor dependent, their proliferation and migration was slowed when cultured at low exogenous EGF concentrations. As a consequence, initially dispersed individuals migrated randomly, but adhered tightly together when encountering another. This behavior persisted for cells with induced expression of Snail, which would otherwise undergo a complete EMT and remain individual when cultured in growth media containing saturating levels of EGF. Over 24 h, these individuals transitioned to multicellular clusters that adopted branching morphologies, since they did not rearrange into more compact morphologies (Fig. 3d). Subsequently, leader cells emerged at the periphery of these branches with transient migration outward, which eventually came into contact with adjacent clusters. Finally, these initially isolated clusters merged together as sparse, space-filling networks. This self-organizing process has remarkable quantitative analogies to the physics of aggregating, non-living colloidal particles, which undergo random walks with irreversible adhesion, and organize into space-filling, fractal-like structures [92], analogous to branching networks of epithelial or endothelial cells.

## Squeezed and stretched: intermediate filaments and EMT

**Fig. 4:**
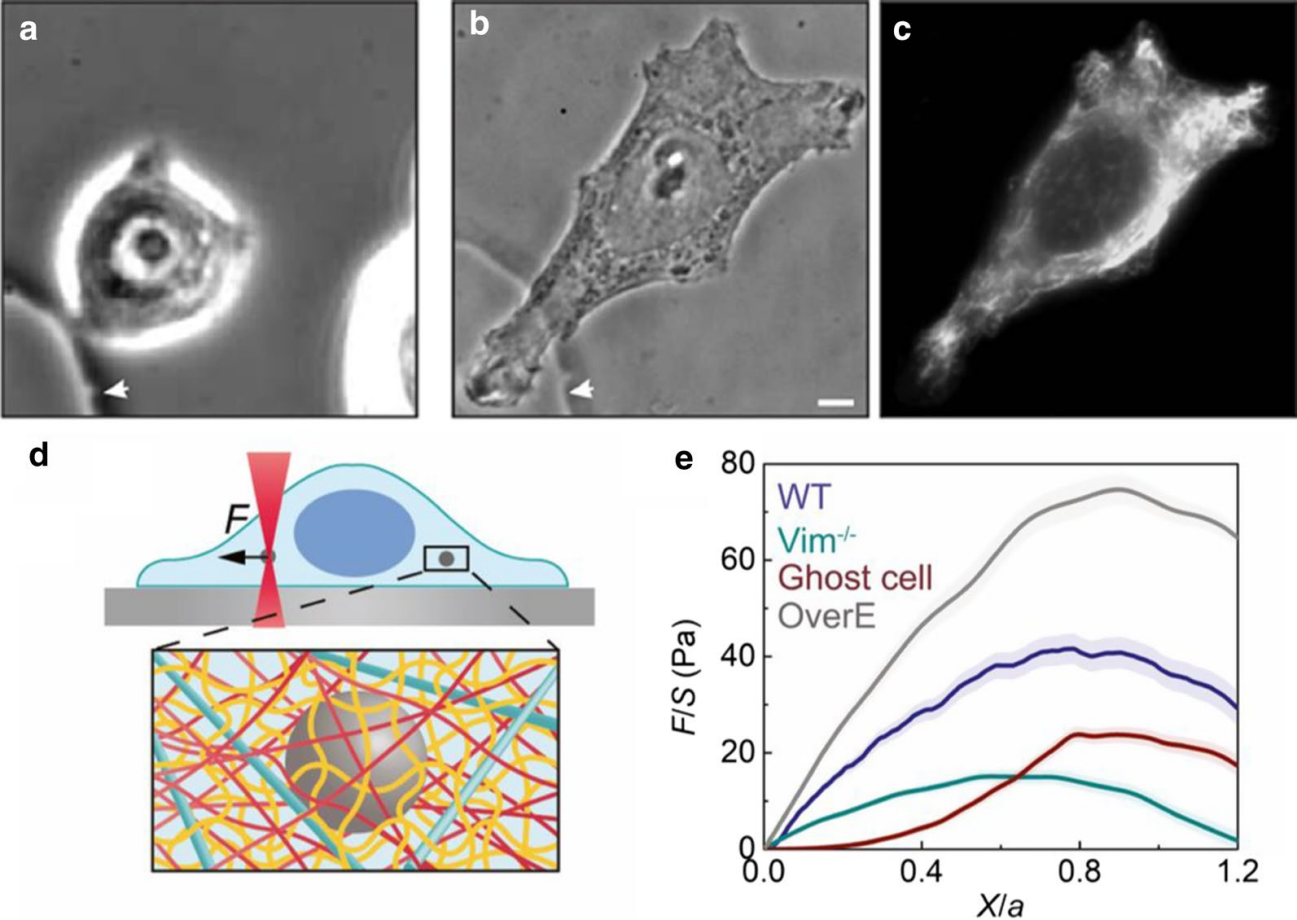
Cell morphology and mechanics are vimentin dependent. **a** Epithelial breast cancer cells (MCF-7) that only express keratin are initially compact and rounded. **b** After microinjection of recombinant vimentin, cells adopt an elongated, mesenchymal-like morphology, with **c** immunofluorescence staining (vimentin). Reproduced from [93]. **d** Optical tweezer measurements manipulate embedded tracer particle to probe local cytoskeletal mechanics. **e** Increasing vimentin expression in mouse embryonic fibroblasts increases mechanical deformability before yielding. Abbreviations denote vimentin knockout (Vim –/–), decellularized vimentin (“ghost cell”) with all other cellular components removed, wildtype (WT), and vimentin overexpression (OverE). Reproduced from [94]

Mendez et al. manipulated vimentin expression within a mammary epithelial cell line (MCF-7), resulting in dramatic alterations of cell morphology and EMT [93]. Luminal-like MCF-7 cells express only keratins and adopt a rounded morphology in 2D culture (Fig. 4a), consistent with an epithelial phenotype. Remarkably, microinjection of recombinant vimentin into these cells was sufficient to cause cellular elongation into a spindle-like morphology (Fig. 4b, c). Moreover, forced transfection of vimentin caused MCF-7 to weaken cell–cell junctions, dispersing as individuals with increased motility. Conversely, RNA silencing of vimentin in mesenchymal breast cancer cell lines (MDA-MB-435) resulted in more compact morphologies consistent with epithelial cells. Analogous results were observed using nocodazole drug treatment to depolymerize microtubules, which in turn reorganized vimentin architecture. Thus, transitions between epithelial and mesenchymal states can occur directly through cytoskeletal organization without manipulation of EMT transcription factors and E-cadherin.

Guo et al. recently used optical tweezers to directly probe vimentin mechanics within live mouse embryonic fibroblasts (mEFs) [95]. Subsequently, Hu et al. found that vimentin positive wild type mEFs exhibited significantly higher strength, stretchability, and toughness relative to vimentin knockouts [94]. Moreover, the presence of vimentin networks decreases the effective cytoskeletal mesh size and thus increases both viscoelastic and poroelastic relaxation times of the cytoskeleton [94, 96]. Indeed, vimentin dominated the mechanical response of mEFs, especially at large deformations ($>50$% strain), while cell mechanics at small deformations was mainly attributed to other cytoskeletal components. This is consistent with macroscopic rheology measurements of reconstituted cytoskeletal networks, where vimentin was much more extensible before failure relative to F-actin or microtubules (Fig. 2d) [40]. These results suggest that vimentin increases the deformability of the cytoskeleton (Fig. 4d, e), and is thus more robust against large deformations. Indeed, these wild type mEFs retained high viability ($>90$%) after being subjected to uniaxial stretch (up to 300% strain), while vimentin knockout fibroblasts exhibited poor viability under comparable strains.

**Fig. 5:**
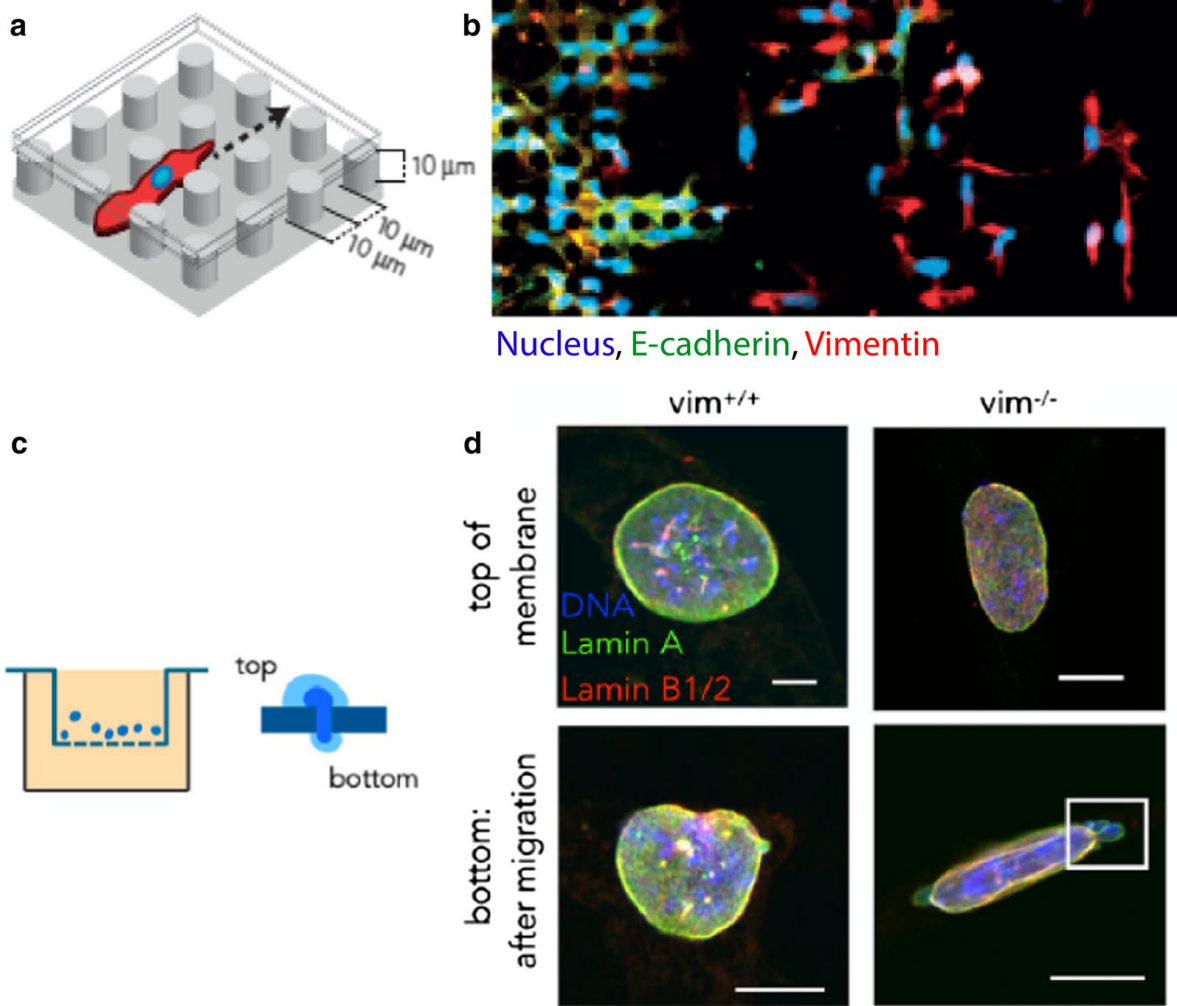
EMT and vimentin in confined spaces. **a** Micropillar arrays for confined migration fabricated using an elastomeric silicone with 10 $\upmu$m spacing and height. **b** Mammary epithelial cells (MCF-10A) migrate as individuals breaking away from a collective front after EMT induction via the Snail transcription factor. Immunofluorescence staining of the nucleus (blue), E-cadherin (green), and vimentin (red). Reproduced from [97]. **c** Transwell (Boyden Chamber) assay for migration through confined pores. **d** Nuclear morphology of wild type and vimentin knockout fibroblasts after traversing membrane. Immunofluorescnece staining of the nucleus (blue), Lamin-A (green), and Lamin B (red). Reproduced from [98]

Microfabricated structures have been used to further investigate cell migration in confined spaces [15]. Wong et al. [97] prepared micropillar arrays with a fibronectin-coated silicone elastomer (poly-dimethylsiloxane), with spacing and height of 10 $\upmu$m (Fig. 5a). Mammary epithelial cells (MCF-10A) with induced Snail expression migrated through these micropillars as individual mesenchymal cells (with vimentin expression), followed by a collective epithelial front (with E-cadherin expression) (Fig. 5b). This narrow 10 $\upmu$m pillar spacing was crucial by limiting cell–cell adhesions, permitting individual mesenchymal cells to detach and disseminate. In comparison, slightly wider spacings resulted in single or multi-file migrations, since epithelial cells had enough room to reorganize and advance without fully breaking cell–cell adhesions. This work was combined with automated single cell tracking to automatically classify subpopulations that exhibited collective or individual migration, which correlated with drug response to targeted inhibitors.

Patteson et al. [98] investigated how vimentin affects confined cell migration across a Transwell membrane with 3-8 $\upmu$m diameter pores, as well as microchannels [99] (Fig. 5c). Vimentin positive wild type mouse embryonic fibroblasts exhibited impaired migration through confined spaces relative to vimentin knockout fibroblasts. However, vimentin knockout fibroblasts suffered increased nuclear damage, with blebbing and DNA double-strand breaks (Fig. 5d). Thus, vimentin mechanically cushions the cell nucleus against compression and nuclear rupture through the presence of a stiff perinuclear shell. This work complements recent results on lamin intermediate filament expression, which also serves to protect the cell nucleus in confined spaces [100, 101]. Overall, these results indicate that vimentin plays an important role in mesenchymal cells both to coordinate cell migration and permit nuclei and cells to undergo large deformations.

## On the tracks: EMT and micro/nano topographies

Classical 2D monolayer culture presents a uniform and flat surface topography that does not spatially bias cell shape or adhesion. In comparison, the extracellular matrix in vivo can be highly fibrillar, with collagen fibers ranging in diameter from 20 to 200 nm [102]. Historically, synthetic nanofibers have been prepared by electrospinning to model such topographies, which extrude viscous polymeric solution from a nozzle under extremely high electric fields and permits limited control over fiber size and organization [103]. Epithelial cells cultured on these fibrillar topographies tend to exhibit elongated morphology, directed migration, and some EMT-associated gene expression [104–107]. Moreover, grooved topographies can also be prepared by controlled “wrinkling” of a stiff coating on a softer substrate [108], which can be reversibly actuated [109, 110].

**Fig. 6:**
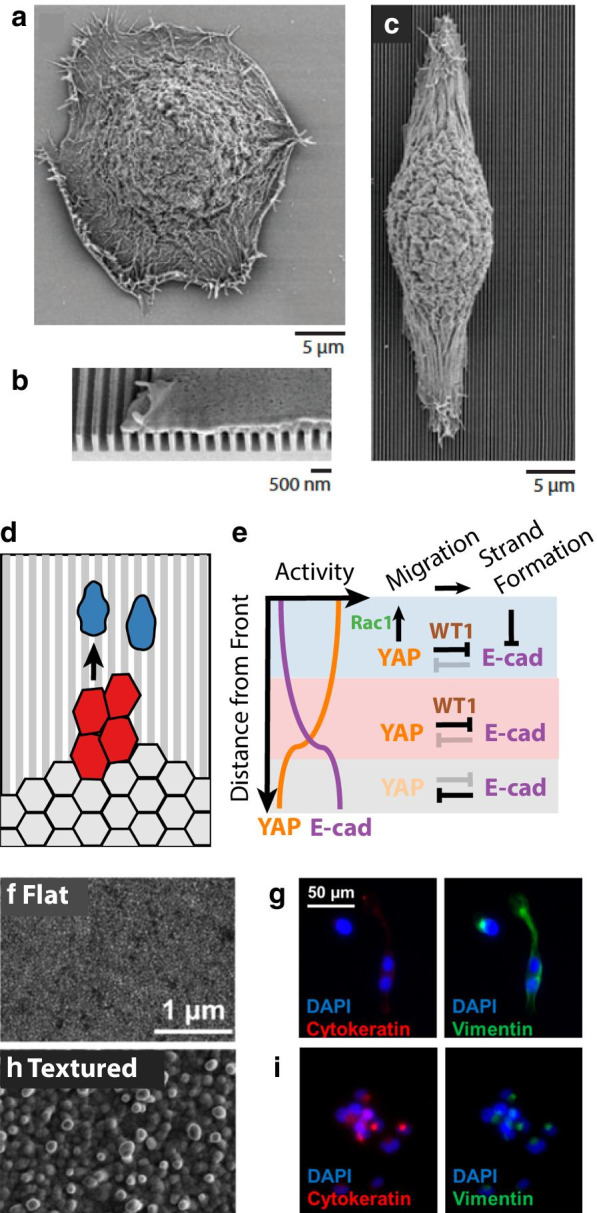
Cellular adhesion on submicron topographies controls EMT/MET. **a** Primary human corneal epithelial cells ordinarily adopt compact, rounded morphologies. **b** Adhesion to grooved topographies with submicron spacing biases focal adhesions along grooves. **c** Epithelial cells subsequently orient and elongate along the groove direction, analogous to an EMT. Reproduced from [111]. **d** Monolayers of canine kidney epithelial cells (MDCK) migrate as multicellular strands (partial EMT) and individuals (complete EMT) on submicron grooved topographies. **e** Crosstalk of YAP and E-cadherin via WT1 regulates partial and complete EMT states. Redrawn from [113]. **f**, **g** Mesenchymal breast cancer cell lines on smooth gold surfaces exhibited elongated morphologies with minimal cytokeratin (red) and strong vimentin (green) expression. **h**, **i**. Mesenchymal breast cancer cell lines on textured gold surfaces exhibited compact, clustered morphologies with strong cytokeratin (red) and weak vimentin (green) expression, indicative of a mesenchymal-to-epithelial transition. Reproduced from [114]

Instead, Texeira et al. [111] patterned submicron grooves with highly controlled geometries by etching silicon wafers, utilizing standard photolithography techniques established for integrated circuit fabrication. Primary human corneal epithelial cells cultured on planar silicon remained rounded and compact (Fig. 6a). However, epithelial cells cultured on nanogrooves exhibited spatially restricted adhesions of filopodia and lamellipodia along the ridge walls (Fig. 6b). As a consequence, these epithelial cells aligned and elongated their cell body along the ridge direction, analogous to an EMT driven by “contact guidance” (Fig. 6c). Subsequently, Ray et al. systematically investigated how different cancer cell lines of varying mesenchymal state were affected by grooved topographies [112]. Interestingly, mesenchymal cells more strongly responded to topography, but isolated epithelial cells without contacts also responded to topography, although in a less pronounced manner. Moreover, epithelial clusters responded to topography only when leader cells extending protrusions were re-directed by nearby topographical boundaries. Thus, cellular transduction of surface topography is mediated by crosstalk with cell–cell adhesions, and is likely to be highly responsive to specific microenvironmental conditions.

Park et al. [113] further investigated how nanotopographical cues affect collective cell migration and EMT. Kidney epithelial cells (MDCK) were cultured as multicellular sheets on grooved substrates. Consistent with previous work, collagen-coated ridges alone are sufficient to induce an EMT phenotype at the advancing margins, with migration velocities similar to TGF-*β* treated cells forced into EMT on flat substrates. The degree of EMT was shown to exist as a gradient in the migrating cell sheet, in which partial to complete EMT is observed in leader cells with few cell–cell contacts, and suppression of EMT is observed in tightly packed cells in the center of the sheet with many neighbors (Fig. 6d). The authors found that the activity of YAP mirrored the distribution of EMT states: YAP was active and localized to the nucleus in leader cells, while it was diffuse in the cytoplasm for cells in the center. Further, authors identified two cross-regulatory networks of YAP that control YAP’s sensitivity to topography (Fig. 6e). First the Wilms tumor protein (WT1), associated with regulating MET and kidney development, was found to be transcriptionally regulated by forming a complex with YAP, which in turn suppressed E-Cadherin, promoting the strongest EMT phenotype in leader cells with few cell–cell contacts. Second, YAP activity upregulated Rac-1 and thus enhanced migration speed in leader cells. These results solidify the notion that pure mechanical cues regulate gene expression: cells with enhanced EMT activity due to micrograte topography exhibit reduced migration capacity when YAP is knocked down. However, when ROCK is also knocked down, Rac-1 mediated migration activity is rescued only in cells on micrograted surfaces, but not flat surfaces. Indeed, the activity of these molecular regulators follows the observed gradient in YAP activity and EMT phenotype.

Wang et al. [114] patterned hierarchical textures in gold using electrochemical deposition techniques, inspired by the fractal-like topography of bone tissue (Fig. 6f, h). Interestingly, the authors found that submicron surface texturing caused mesenchymal breast and prostate cancer cell lines to revert to an epithelial phenotype, representing a reverse mesenchymal-to-epithelial transition (MET). In response to nanotopography and limited cell-substrate adhesions, cells exhibited rounded morphology, reduced proliferation observed via Ki-67, as well as increased junction formation indicated by increases in ICAM1, E-Cadherin, and keratin expression, while cells on bare gold substrates maintained a mesenchymal phenotype (Fig. 6g, i). Further, cells interacting with nanostructures displayed down-regulated RhoA and Cdc42, whereas Rac1 maintained high levels of expression compared to cells residing on flat gold substrates (Fig. 6h, i). A global decrease of mesenchymal markers was observed, including N-Cadherin, vimentin, ZEB1 and ZEB2. These effects corresponded to functional differences in migration capacity, in which MET’d cells displayed a decreased wound-healing response and diminished migration in Transwell chamber assays. Cells were then re-cultured on flat tissue culture plastic (TCP) for 7 days following the initial 7-day incubation on nanostructures. These re-cultured cells maintained their rounded morphology and expression of junction proteins, but displayed more active proliferation, indicating growth arrest was transient. Finally, the authors showed that the nanostructure-mediated MET process is transduced by downregulating glycogen synthase kinase-3 (GSK-3) phosphorylation, which decreased the expression of Snail. Overall, these findings demonstrate that diminished cell–matrix adhesions via nanotopographical cues may be compensated for by enhanced cell–cell adhesions, which would promote a clustered epithelial cell state.

## Enter the matrix: EMT in 3D

Epithelial cells embedded within a compliant tri-dimensional (3D) matrix recapitulate tissue architecture and invasive behaviors observed in vivo. In particular, single cells cultured in reconstituted basement membrane (i.e. Matrigel) or collagen I will organize into multicellular clusters with differentiated architectures, strong cell–cell junctions, as well as hollow lumens that mimic ducts or glands [13, 115]. Notably, an increase in matrix stiffness is sufficient to drive the disorganization and local dissemination of multicellular clusters via integrin-mediated signaling [116, 117]. Indeed, cancer cells exhibit cytoskeletal polarization and directional migration in response to diverse “-taxis” phenomena. Guiding cues driving these behaviors include soluble signals (i.e. chemotaxis) [118], bound chemo-attractants/repellents (i.e. haptotaxis), stiffness gradients (i.e. durotaxis) [119], local topography or ECM density (i.e. topotaxis) [120], interstitial flow (i.e. rheotaxis) [121, 122], and applied electrical fields (i.e. electrotaxis) [123]. Reconstituted biomaterials have revealed new insights into these processes, as well as the importance of matrix remodeling. In particular, matrix metalloproteinase (MMP) activity is crucial for multicellular (collective) invasion to occur through progressively widening pores [124]. Indeed, 3D traction force microscopy has revealed strong front/back tractions for individual mesenchymal cells, particularly in fibrillar matrix [125–127]. However, MMP activity may be dispensable for individual migration, since cells can utilize a propulsive “amoeboid” mode to squeeze through the matrix, ultimately limited by nuclear deformability and matrix pore size [128].

Transitions from multicellular clusters to collective or individual invasion via EMT-associated signaling can occur in response to both soluble and matrix cues. Notably, Hay’s initial demonstration of EMT was induced solely by culture of corneal epithelial cells in fibrillar collagen I matrix [4], which has been corroborated by Reinhart-King’s group using mammary epithelial cells (MCF-10A) [129]. Jing Yang’s group has shown that stiff substrates (with an overlay of Matrigel) can activate TWIST in mammary epithelial cells (MCF-10A), resulting in enhanced dissemination [130, 131]. Friedl’s group has shown that hypoxic conditions can also induce HIF1 and downstream EMT signaling, which unexpectedly results in amoeboid rather than mesenchymal migration in murine breast cancer cells (4T1) [132]. EMT can also be induced by exogenous treatment with growth factors (e.g. EGF, HGF, TGF-*β*) in various epithelial cell lines, resulting in collective invasion and branching morphogenesis [133–137]. Nevertheless, EMT induction is not accompanied by invasion in synthetic 3D matrices that resist MMP degradation [138, 139]. It should be noted that EMT induction in 3D matrix also depends sensitively on cell–cell junctions. Indeed, Snail induction is impeded in multicellular clusters of mouse mammary epithelial cells with established apicobasal polarity [140]. Moreover, Ewald’s group showed that Twist induction in primary mouse mammary epithelial organoids results in collective invasion that retains E-cadherin expression [141]. Thus, elucidating EMT in 3D matrix requires careful consideration of tissue architecture and microenvironmental cues.

**Fig. 7:**
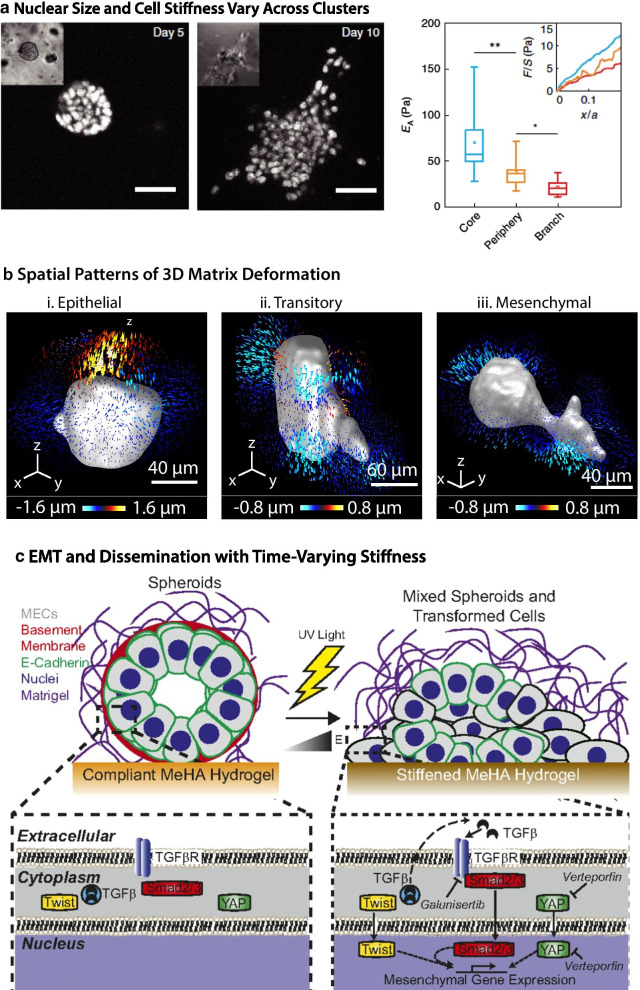
Collective invasion and EMT in 3D matrix. **a** Multicellular clusters of mammary epithelial cells (MCF-10A) cultured in a alginate-Matrigel mixture first form spherical acini over 5 days, but subsequently disorganize and disseminate over 10 days. Remarkably, nuclear and cellular volume increase at the periphery, corresponding to softer intracellular stiffness (right). Reproduced from [142]. **b** Multicellular clusters also exert spatially non-uniform patterns of protrusive and contractile tractions for epithelial, transitory (EMT), and mesenchymal states induced via Snail. Reproduced from [143]. **c** Multicellular clusters respond to dynamically stiffened substrates via dissemination and EMT activation via Twist, TGF-*β*, and YAP signaling. Reproduced from [144]

Han et al. [142] investigated the spatial patterns of cell size and stiffness in an invasive multicellular cluster, or cancer organoid, embedded within a composite Matrigel-alginate hydrogel. This composite matrix was engineered to mimic the mechanical properties of breast carcinoma in vivo (shear modulus $\sim$300 Pa, comparable to tumor ECM), resulting in the disorganization and dissemination of mammary epithelial cells (MCF-10A) from 5 to 10 days in culture (Fig. 7a). These clusters exhibited considerable spatial heterogeneity in cell size and volume. In particular, cells in the central core remained compact with increased stiffness, while cells at the periphery exhibited increased nuclear volume with decreased cell stiffness (Fig. 7a). Indeed, cells used gap junctions to dynamically manipulate their size by regulating fluid and ion transport. Pharmacological treatments to perturb cell size or stiffness impeded collective invasion at the periphery, but had limited effect on cell proliferation. Moreover, tumor biopsies from breast cancer patients exhibited a similar pattern of larger nuclear sizes at the invasion front, suggesting that differences in cell volume may be important for invasion in vivo. Nevertheless, it should be noted that nuclear and cell morphology are highly dysregulated during tumor progression, which may confound some of these trends.

Leggett et al. [143, 145] investigated the spatial patterns of 3D matrix deformations of multicellular clusters (MCF-10A) at varying stages of inducible Snail expression within a composite silk fibroin-collagen I hydrogel. Epithelial clusters (with no Snail induction) exhibited a spherical morphology and applied both protrusive and contractile tractions to the surrounding matrix (Fig. 7b, i). “Transitory” clusters with Snail induction after embedding extended invasive protrusions and spatially distributed contractile tractions (Fig. 7b,ii). Finally, mesenchymal clusters with Snail induction before embedding were highly elongated with localized contractile tractions in a few locations (Fig. 7b,iii). These spatially non-uniform protrusive and contractile tractions were used to classify clusters into three distinct mechanophenotypes that exhibited 70–75% agreement with the experimental treatment condition, which is plausible given the intrinsic heterogeneity of these cells. These mechanophenotypes could be perturbed using pharmacological treatment against cytoskeletal or EMT associated pathways. For example, sublethal treatment with the microtubule stabilizing agent Taxol (Paclitaxel) shifted towards more mesenchymal states, so that initially epithelial clusters appeared more transitory, etc. In comparison, treatment with the epidermal growth factor receptor (EGFR) inhibitor Gefitinib had minimal effect on initially epithelial or mesenchymal clusters, but caused the initially transitory clusters to exhibit epithelial or mesenchymal mechanophenotypes. It should be noted that this technology was implemented on a 96 well plate, enabling higher throughput measurements of cell–matrix interactions across different drug treatment conditions.

Ondeck et al. investigated multicellular dissemination and EMT using biomaterials with time-varying stiffness [144]. Their system utilized an underlying substrate consisting of methacrylated hyaluronic acid (HA), which was partially crosslinked using ultraviolet light to be “soft,” (100 Pa) and could subsequently be “stiffened” (3000 Pa) through additional ultraviolet exposure (Fig. 7c). These substrates were coated with collagen I, and further overlaid with mammary epithelial cells (MCF-10A) and Matrigel. Typically, single cells formed compact acini on soft substrates, but disseminated as elongated mesenchymal cells on stiffened substrates. Interestingly, the extent of dissemination depended on the duration of culture on soft substrates. For example, cells cultured for shorter durations on soft substrates exhibited complete dissemination after stiffening. However, some cells cultured for extended durations (10 days) on soft substrates remained as compact acini after stiffening. This heterogeneous response was recapitulated when cells were sorted as individuals or acini, then re-cultured on a new biomaterial, suggesting a lack of mechanical “memory.” Immunofluorescence staining suggested that these heterogeneous responses were driven by a combination of paracrine signaling through TGF-*β*/SMAD, as well as YAP localization. The combined inhibition of these two pathways through Galunisertib and Verteporfin impeded migration and rescued epithelial acini even on stiffened substrates. It should be noted that this overlay geometry does not fully confine cells relative to complete embedding in 3D matrix, which has been reported to limit YAP activation [146]. Nevertheless, the use of time-varying biomaterials represents an elegant approach to investigate phenotypic plasticity and EMT.

Finally, Ranamukhaarachchi et al. showed that mesenchymal breast cancer cell lines could revert to epithelial biomarker expression within a collagen I matrix with compacted fibrils [147]. Briefly, collagen I networks were polymerized in the presence of an inert macromolecular crowding agent (polyethylene glycol) at varying concentrations. The crowding agent decreased collagen I fiber length and degradability, while leaving porosity and stiffness roughly comparable to the untreated controls. As a consequence, adenocarcinoma cells (MDA-MB-231) formed connected multicellular tubule architectures in collagen I networks formed with the crowding agent. Indeed, at very high concentrations of crowding agent, these cells formed epithelial acini with hollow lumens and cell–cell junctions (e.g. PECAM1, ICAM1). This is particularly noteworthy since MDA-MB-231 is a highly mesenchymal cell type with minimal E-cadherin junctions, which is difficult to revert to an epithelial phenotype. This result has analogies with the MET observed by Wang et al. [114] on nanostructured topographies with reduced cell-substrate interactions, illustrating the role of matrix architecture on epithelial plasticity.

## Discussion

### Precision measurement and engineering of the local mechanical microenvironment

EMT is associated with the dynamic acquisition of an elongated, migratory phenotype, which is mediated by a redistribution of cell–matrix adhesions [22]. In order to resolve these cell state transitions, there is an increased need to map molecular and subcellular changes within a heterogeneous population over space and time [148]. Bioengineering approaches allow direct visualization of how cells apply forces to a compliant biomaterial, both on planar 2D substrates [86] as well as tri-dimensional 3D matrix [143], which we summarize in Table 1. In 3D, epithelial cells are initially compact and round with uniformly distributed tractions around the periphery. Activation of EMT pathways drives protrusion formation around the cell periphery, as well as localizing tractions at the front and back, ultimately resulting in a spindle-like morphology. We envision that the sensitivity of these mechanical measurements could be further improved using emerging imaging modalities to resolve tracer particles at high density (e.g. super-resolution microscopy) [149], or directly resolve fibrillar matrix proteins (e.g. second harmonic generation) [150]. Moreover, an improved understanding of the mechanical response (i.e. constitutive equations) of extracellular matrix is needed, since these fibrous networks also exhibit strain-stiffening [36] and can be extensively remodeled by cells [5].

Biomimetic materials can also be engineered to shape cell–matrix adhesions in order to perturb EMT. Individual cells cultured on topographical patterns such as submicron grooves will align and elongate [111, 112], while leader cells can emerge from the collective migration front of multicellular sheets [113]. Similarly, clusters can disorganize and disseminate in stiffer and more fibrillar 3D matrix [116, 117], which mimics the desmoplastic stroma in malignant tumors [5]. Conversely, impeded cell–matrix adhesions on fractal-like topographies [114] or degradation-resistant collagen I [147] can force mesenchymal cells to revert to an epithelial state. Overall, Hippo/YAP signaling appears to play an important role in transducing mechanical cues towards EMT (in conjunction with other pathways) [29], although this may be cell-type dependent. An important approach for elucidating time-dependent phenotypic plasticity is to design new biomaterials whose properties can be dynamically tuned by external stimuli [144]. Photopatterning of 3D biomaterial topography and surface chemistry with improved spatial resolution could further shape cell behavior [151], which can be combined with the higher resolution measurement techniques described previously. There is also great interest in decellularized matrix prepared by removing living cells from animal or human tissue, which preserves much of the biochemical and structural complexity of ECM in vivo (see review in [152]). Decellularization techniques have also been applied to ECM deposited by cancer-associated fibroblasts, which can significantly impact cancer cell invasion, proliferation, and gene expression [153, 154].

**Table 1:** Comparison of EMT/cell migration assays

Technique	Method	Comments	References
Cell scattering assay (2D)	Plate cells at subconfluent density on planar 2D substrate; use time-lapse imaging to visualize how multicellular clusters disperse into individuals, particularly in response to growth factor stimulation (e.g. HGF, EGF)	Straightforward experimental technique, which can be integrated with 2D traction measurements. However, it is difficult to control cluster size, and cell migration occurs in random directions	[83–87, 89]
“Wound Healing” Assay (2D)	Plate cells at confluent density on planar 2D substrate; observe how these monolayers collectively migrate into unoccupied regions, either by mechanically “scratching” to remove cells or by removing a barrier	Also straightforward experimentally, and cells migrate in a preferred direction, which may not require time-lapse imaging. However, “scratching” can leave cell debris or damage soft substrates, and this assay biases towards collective migration	[9, 88, 90, 91]
Micropatterned substrates (2.5D)	Analogous to cell scattering or wound healing assays, but incorporating topographical features on the substrate	Mimics ECM architecture and can affect directional migration via contact guidance, as well as EMT/MET. However, requires some expertise and specialized equipment to fabricate	[15, 102–104, 107–111, 113, 114]
Transwell/Boyden chamber	Plate cells on top of a porous plastic membrane, count the number of cells that migrate across membrane to the other side. Can include a gradient of chemotactic factors across membrane, as well as an ECM coating on the top	Standard assay that can be easily quantified. However, difficult to image cell migration in this vertical geometry, and bias towards individual migration since cells must squeeze through small pores	[10, 98, 100, 128]
Confined microchannels	Load cells into a microfluidic device; use time lapse-imaging to visualize how cells migrate through confined geometries such as channels or micropillar arrays. Can also include a gradient of chemotactic factors along the channel	Mimics ECM porosity and can evaluate nuclear and cellular deformability in confinement. However, the elastomeric silicones used tend to be much stiffer than ECM. Also requires some expertise and specialized equipment to fabricate	[15, 97, 99, 101]
Individual invasion in 3D matrix	Embed single cell dispersion into a crosslinked 3D matrix; use time lapse-imaging to visualize how cells migrate and undergo EMT	3D matrix can be tuned with varying biochemical composition, matrix stiffness, and porosity to mimic ECM architecture in vivo. However, cell migration occurs randomly and will vary with the specific matrix conditions selected. Moreover, preparing 3D matrix requires specialized training and experimental optimization	[12, 14, 24, 28, 47, 50, 98, 105, 115–117, 125–131, 133–135, 138–140, 142, 143, 145–147]
Spheroid/organoid invasion in 3D matrix	Generate multicellular spheroids or isolate tumor fragments and embed into a crosslinked 3D matrix; observe spheroid/tumor size and outward invasion	Diverse approaches to prepare larger tissues with multiple cell types and investigate collective invasion; however, transferring spheroids and tissue into 3D matrix must be done carefully and can be tedious. 3D matrix properties are tunable, requiring additional optimization for larger spheroids that can settle to the well bottom	[4, 11, 132, 136, 141, 144]
Animal models of cancer (mouse, zebrafish, chick CAM assay)	Generate fluorescently labeled or luciferese-labeled tumors in an animal model; implant cancer cell lines into immunodeficient organisms, or use a genetically engineered cancer model, anesthetize and image over time using intravital microscopy, or bioluminescence imaging	Can be used to monitor tumor growth, invasion, intravasation/extravasation, transmigration, and EMT in an in vivo tumor microenvironment. However, immunodeficient models and species differences (host vs. humans) may complicate interpretation of the results	[155–158]
Histology	Fix and stain thin sections of tissue from a patient biopsy, tumor tissue from an animal, or patient-derived xenograft	Allows direct measurement of invasive cancer cells in a human patient tumor, but occurs as a two-dimensional snapshot	[159]

Cell–cell adhesions (e.g. E-cadherin) mediate transitions between anchored tissues, collectively migrating groups, and individual mesenchymal phenotypes [3]. Experiments on planar 2D substrates have shown that increased motility in response to HGF is sufficient to rupture cell–cell junctions without associated downregulation of E-cadherin [85]. Similarly, slowed cell motility in reduced EGF results in multicellular cluster formation and collective behaviors [89], reminiscent of an MET. Existing traction force microscopy methods are only capable of inferring cell–cell forces from cell–matrix tractions for small cell clusters, where all cells are in contact with the underlying 2D substrate. It remains challenging to measure forces deep within 3D clusters, and may require the use of molecular force sensors or embedded probe particles that are compliant [160]. Nevertheless, there is increasing evidence that extensive cell–cell contacts (i.e. near confluency) that maintain apicobasal polarity restrict EMT activation, both in 2D [161, 162] and in 3D [140]. Biomaterial patterning techniques may also be employed to define the shape of multicellular clusters, in order to indirectly modulate the configuration of cell–cell adhesions for any given cell [163, 164].

### Probing intracellular mechanics and the cytoskeleton

EMT is associated with the loss of keratin and the gain of vimentin intermediate filaments, although the functional role of these cytoskeletal proteins is still being investigated. Remarkably, microinjection of vimentin into mammary epithelial cells (MCF-7) is sufficient to drive morphological elongation, analogous to EMT [93]. The presence of vimentin within the cytoskeleton also protects the cell against externally applied stretch [94], and provides structural integrity to limit nuclear damage as cells traverse through confined spaces [97–99]. An ongoing challenge for this field is to mechanically probe subcellular mechanics, which are confounded by different contributions of thermal forces and the activity of molecular motors [165, 166]. Indeed, Gupta et al. showed that cell mechanical properties can vary considerably across transverse and longitudinal directions for elongated cells confined to micropatterns on planar 2D substrates [167]. Intracellular mechanics are even more challenging to probe in situ for cells embedded in 3D matrix, since they are inaccessible to most mechanical characterization techniques based on direct contact.

Guo et al. recently demonstrated that intracellular stiffness is inversely proportional to cell and nuclear size [66]. In particular, mammary epithelial cells at the core of a multicellular cluster were smaller and stiffer, while cells at the periphery were larger and softer [142]. Qualitatively similar trends were observed for nuclear size in breast tumors from human patients. This phenomenon will require further validation in human patients, since nuclear and cell morphologies are typically dysregulated during tumor progression. Nevertheless, tracking how these morphological features vary in space and time could give new insights into cell stiffness. More generally, the improved control and visualization of cytoskeletal structure can be coupled with functional assays of cell stiffness and tractions for both individuals and multicellular groups. An interesting possibility is the use of machine learning and convolutional neural networks to profile distinct patterns of cytoskeletal organization that correlate with enhanced invasion and EMT [168], both in these bioengineered models as well as for intravital imaging and patient histology. Moreover, computational modeling can approximate single cells as discrete “agents” with some representation of intracellular signaling networks, along with cell and matrix mechanics (see recent reviews in [169, 170]). These agents can further interact with a surrounding microenvironment that models hypoxia, chemotactic gradients, etc. at coarser length scales (i.e. a continuum treatment using partial differential equations), for better computational efficiency (see review in [171]). Thus, higher resolution experimental measurements in space and time can be refined by computer vision and inform quantitative simulations to make testable predictions about cancer cell biology.

### EMT in cancer metastasis

EMT and vimentin expression play an important role in directed cell migration, which is a crucial step in tumor invasion [8]. In particular, EMT is often observed at invasion fronts in vitro [88, 97, 113, 142], consistent with in vivo observations of EMT at the periphery of human patient tumors [172]. Vimentin plays an important role in coordinating focal adhesions and enhancing actomyosin contractility [173], as well as invadopodia to degrade the basement membrane [82]. However, the role of vimentin and EMT for cancer cells that intravasate into the bloodstream and extravasate into the metastatic site remains unresolved. It is likely that vimentin can protect the cell nucleus as cells traverse confined spaces within capillaries, consistent with measurements of migratory cells in microchannels [98, 99]. Vimentin also supports tubulin-based membrane protrusion called “microtentacles” that facilitate cancer cell adhesion and arrest on the vasculature [174]. However, EMT has not been proven to be essential for metastatic cancer in humans [7].

Although transitions between epithelial and mesenchymal phenotype are tightly controlled in development and wound healing, it is conceivable that a complete spectrum between epithelial and mesenchymal states occurs in tumor progression (i.e. cancer as a caricature of development). Yu et al. characterized circulating tumor cells (CTCs) from breast cancer patients using RNA-in situ hybridization, showing distinct states with only epithelial transcripts, “hybrids” with both epithelial and mesenchymal transcripts, or only mesenchymal transcripts [175]. Biomarkers of this “hybrid state” could manifest as co-expression of vimentin and keratin intermediate filaments. Pioneering work by Hendrix and coworkers investigated genetic co-expression of keratin (K8 and K18) with vimentin in breast cancer and melanoma cell lines, showing some increase in 3D invasion due to altered integrin expression [176, 177]. Recently, Pastushenko et al. identified a “partial EMT” state that expresses both vimentin and keratin-14 (but not EpCAM), with increased metastatic potential in mouse models of melanoma (KRas^LSL-G12D^p53^fl/fl^) and breast cancer (MMTV-PyMT) [178]. Indeed, a subpopulation of tumor cells has been previously observed with co-expression of vimentin and keratin in colorectal cancer patients (“tumor budding”) [179], as well as from metastatic cancer cells in breast cancer patients [180, 181].

CTCs can be isolated from peripheral blood as multicellular clusters, raising the intriguing possibility is that groups of cells with varying epithelial or mesenchymal states can further cooperate at varying stages of metastasis. Aceto et al. showed that CTC clusters exhibited 50-fold enhanced metastatic potential relative to single CTCs in mouse models, suggesting that disseminated micrometastases are often polyclonal [182]. Cheung et al. [183] have highlighted the role of keratin-14 in (vimentin negative) epithelial leader cells as well as adhesion between CTCs within clusters [184] in a genetically engineered mouse model of breast cancer (MMTV-PyMT). Overall, the functional role of keratin-14 remains poorly understood, but could conceivably substitute for vimentin to coordinate cell migration and protect the cell nucleus, and merits further investigation at single cell resolution. More nuanced genetic manipulation of signaling pathways in small animal models may enable deeper understanding of functional phenotypes, which can be corroborated back to human patient data.

EMT may occur transiently during different states of tumor progression. In particular, EMT is associated with early dissemination in a number of genetically engineered mouse models [185–187]. One potential explanation is that EMT may be advantageous for tumor cells in inhospitable microenvironments, particularly to establish a pre-metastatic niche [188], evade immune cells [189, 190], or resist drug treatment. Indeed, Fischer et al. and Zheng et al. have reported that EMT is dispensable for metastasis in mouse models but associated with chemoresistance [191, 192]. Similarly, Yu et al.  observed more mesenchymal states and CTC clusters as patients exhibited increased drug resistance. Quantitative profiling of cell morphology, EMT biomarker expression, and mechanophenotype have been used to capture population level heterogeneity and plasticity associated with EMT in vitro [143, 162], and such methods may be useful to interrogate patient samples to guide precision medicine in the future. For instance, Navas et al. [193] designed a quantitative immunofluorescence assay to evaluate the EMT status of patient samples, which revealed a high degree of diversity across patients and carcinoma type. Notably, some patients with advanced disease exhibited highly heterogeneous mixtures of epithelial, partial EMT, and mesenchymal subpopulations, the abundance of which tended to shift after drug treatment toward an increasing mesenchymal fraction. Nevertheless, tumor cells with mesenchymal biomarkers are relatively uncommon at metastatic sites, which could occur since they are outcompeted by faster proliferating epithelial cells once selection pressure is removed. Alternatively, it has been proposed that tumor cells can undergo a reverse MET at a metastatic site, which remains challenging to experimentally verify [7].

Ultimately, improved temporal resolution of single cell states at varying steps of the metastatic cascade are required to elucidate the role of EMT. Intravital microscopy has emerged as a powerful approach for visualizing fluorescently labeled tumor cells within an in vivo tumor microenvironment (reviewed in [156]). In mice, surgery is performed to generate skin-flaps for short-term time-lapses or optical imaging windows for long-term studies to enable intravital microscopy [194]. Alternatively, spontaneous and experimental metastasis assays may be conducted in zebrafish or the chick embryo (chick chorioallantoic membrane assay, “CAM assay”), which provide a more facile and scalable approach for monitoring tumor-stroma interactions [158]. EMT and individual invasion are frequently observed in xenografts and genetically engineered mouse models [195–198], although increased collective invasion has been observed by implantation of multicellular spheroids [199] or organoids [184]. Thus, EMT and invasion phenotype may also vary with cell–cell and cell–matrix interactions in vivo [200], as well as with tissue origin, necessitating more nuanced phenotypic and genetic definitions that can be systematically and rigorously tested [2]. Rigorous validation of cell migration and EMT in bioengineered systems against animal models and patient data should occur not only for genetic and transcriptional profiles, but also functional phenotypes as well.

## Conclusion

Bioengineering approaches enable new insights into EMT and the cytoskeleton through a combination of higher resolution measurement and highly consistent assay geometries. In this review, we highlight our selection of recent results that highlight new capabilities and address unresolved questions in this field. First, cells cultured on planar substrates exhibit coordinated behaviors, with leader cells emerging through EMT-like processes. Second, localized probing of vimentin networks reveals that they enhance the stretchability of cells under large deformations. Indeed, cells that express vimentin can better protect their nucleus and squeeze through highly confined spaces during migration or proliferation. Third, cells cultured on topographically patterned features adhere in a spatially asymmetric fashion, resulting in a reorganization of the cytoskeleton and cell–cell adhesions. Highly aligned features typically result in cell elongation and directional migration along these “tracks,” analogous to EMT. However, fractal-like topographies that limit cell–matrix adhesions can drive mesenchymal cell types to undergo an MET and express epithelial biomarkers. Finally, multicellular clusters cultured in compliant tri-dimensional matrix can disorganize and disseminate with spatially heterogeneous cell stiffness and tractions. These collective behaviors are spatially coordinated across the population by biochemical and mechanical signals. Nevertheless, when matrix remodeling is impeded, mesenchymal cells can undergo an MET and form compact epithelial acini. Overall, these technologies could be utilized for rapid measurements of patient samples (i.e. organoids), mapping tumor transitions at single cell resolution over space and time, which cannot be directly resolved in human patients. Moreover, biomimetic systems enable fundamental insights into how cells behave in well controlled physical microenvironments, and could test drug response at higher throughput than xenograft models. Ultimately, these new technological capabilities will be essential to reveal exceptional cells and rare events that underlie both EMT and tumor metastasis in human patients.

## Abbreviations

**2D**: Two dimensional
**3D**: Three dimensional
**APC**: Adenomatous polyposis coli
**CAM**: Chorioallantoic membrane
**CTC**: Circulating tumor cell
**DIA1**: Formin diaphanous 1
**EGF**: Epidermal growth factor
**EMT**: Epithelial-mesenchymal transition
**GSK-3**: Glycogen synthase kinase 3
**HA**: Hyaluronic acid
**HGF**: Hepatocyte growth factor
**ICAM1**: Intercellular adhesion molecule 1
**LIMK**: LIM kinase
**MDCK**: Madin-Darby canine kidney
**mEF**: Mouse embryonic fibroblasts
**MET**: Mesenchymal-epithelial transitions
**MMP**: Matrix metalloproteinase
**PECAM1**: Platelet and endothelial cell adhesion molecule 1
**ROCK**: Rho-associated Kinase
**TAZ**: Transcriptional coactivator with PDZ-binding motif
**TGF**-*β*: Transforming growth factor beta
**YAP**: Yes-associated protein
**ZEB1/2**: Zinc finger E-box-binding homeobox 1/2
